# Impact of agronomy practices on the effects of reduced tillage systems on CH_4_ and N_2_O emissions from agricultural fields: A global meta-analysis

**DOI:** 10.1371/journal.pone.0196703

**Published:** 2018-05-21

**Authors:** Jinfei Feng, Fengbo Li, Xiyue Zhou, Chunchun Xu, Long Ji, Zhongdu Chen, Fuping Fang

**Affiliations:** China National Rice Research Institution, Hangzhou, China; Tennessee State University, UNITED STATES

## Abstract

The effect of no- and reduced tillage (NT/RT) on greenhouse gas (GHG) emission was highly variable and may depend on other agronomy practices. However, how the other practices affect the effect of NT/RT on GHG emission remains elusive. Therefore, we conducted a global meta-analysis (including 49 papers with 196 comparisons) to assess the effect of five options (i.e. cropping system, crop residue management, split application of N fertilizer, irrigation, and tillage duration) on the effect of NT/RT on CH_4_ and N_2_O emissions from agricultural fields. The results showed that NT/RT significantly mitigated the overall global warming potential (GWP) of CH_4_ and N_2_O emissions by 6.6% as compared with conventional tillage (CT). Rotation cropping systems and crop straw remove facilitated no-tillage (NT) to reduce the CH_4_, N_2_O, or overall GWP both in upland and paddy field. NT significantly mitigated the overall GWP when the percentage of basal N fertilizer (P_BN_) >50%, when tillage duration > 10 years or rainfed in upland, while when P_BN_ <50%, when duration between 5 and 10 years, or with continuous flooding in paddy field. RT significantly reduced the overall GWP under single crop monoculture system in upland. These results suggested that assessing the effectiveness of NT/RT on the mitigation of GHG emission should consider the interaction of NT/RT with other agronomy practices and land use type.

## Introduction

Agriculture is a main source of anthropogenic greenhouse gas (GHG) emissions [1], contributing 47% and 76% of CH_4_ and N_2_O emissions, respectively. Agricultural practices (e. g. soil tillage, fertilizer, and irrigation) play an important role in regulating the microbial process of CH_4_ and N_2_O production in agricultural soil [2]. Detailed knowledge of the effects of agronomical options on GHG emissions is imperative for the recommendation of low emission practices.

Reduced (RT) and no tillage (NT) are widely recommended in world crop production to improve soil structure, reduce soil erosion, and enhance soil organic matter as compared with conventional tillage (CT). However, the effect of NT/RT on climate change mitigation has been intensively debated because of the substantial inconsistency in individual field experiments [3–5]. Previous studies have demonstrated that NT significantly reduced [6], increased [7] or did not affect [8] CH_4_ emission from soil, compared with that of CT. Similarly, a decrease [9], increase [10], or insignificant change [11] of N_2_O emission was observed in response to NT. In addition, the effects of NT on CH_4_ and N_2_O emissions were usually inconsistency. For instance, a previous study reported that NT significantly reduced CH_4_ emission in paddy field compared with CT, but increased N_2_O emission [12]. Similarly in the uplands, NT significantly reduced not only N_2_O emission but also CH_4_ uptake [13]. The trade-off relationship may offset the effect of NT on GHG mitigation. The highly diverse results from individual studies are unlikely to reveal a general pattern of soil tillage on GHG mitigation. Although some studies have been conducted to evaluate the effect of NT or RT on GHG mitigation, they focused only on CH_4_ or N_2_O emissions [14–15]. The integrated effects of RT or NT on the total GWP of CH_4_ and N_2_O emissions has not been well documented.

Soil tillage can affect several soil properties (e.g. soil bulk density, temperature, moisture, and the vertical distribution of crop residue) that influence the production and emission processes of CH_4_ and N_2_O [16–18]. Even more complicatedly, some effects of tillage on CH_4_ and N_2_O emissions function in potentially contrasting ways, making it difficult to predict the effect of a tillage practice on GHG mitigation [14,19]. For example, in paddy fields, NT can increase CH_4_ oxidation by improving the soil structure and decreasing the disturbance on the niche of the Methanogenic bacteria [20–21]; whereas, NT tended to increase the soil organic matter, which facilitated the increase in CH_4_ emission [22]. The integrated effect of NT on CH_4_ and N_2_O emissions was highly dependent on the climatic conditions, soil properties, and agricultural practices. Van Kessel et al. [14] reported that dry climatic conditions were conducive for NT/RT to reduce N_2_O emission based on a global meta-analysis of NT/RT on N_2_O emission in uplands. Zhao et al. [15] reported that the inhibition effects of NT on CH_4_ or N_2_O emissions were negatively correlated with temperature, precipitation, and soil pH by synthesizing the experimental results in China. Besides the climate and soil factors, the interaction of tillage with other agronomy options on CH_4_ and N_2_O emissions was also observed in previous field experiments [23–24]. Van Kessel et al. [14] assessed the interaction of fertilizer application depth with tillage and reported that NT/RT performed better on the mitigation of N_2_O emission when N fertilizer was placed ≥5cm rather than < 5cm. However, the interaction effect of other options (e.g., cropping system and irrigation) with NT/RT is still unclear. A better understanding of the interaction of agronomy practices with NT/RT will be beneficial to determining the best management practices for NT/RT to mitigate CH_4_ and N_2_O emissions in agricultural fields.

Therefore, the main objectives of this meta-analysis were: 1) to quantitatively summarize the effects of NT/RT on the total GWP of CH_4_ and N_2_O emissions and 2) to investigate the effects of cropping system, crop residue management, fertilizer split application, irrigation and tillage duration on the effectiveness of NT/RT.

## Materials and methods

### Data sources

**Fig 1:**
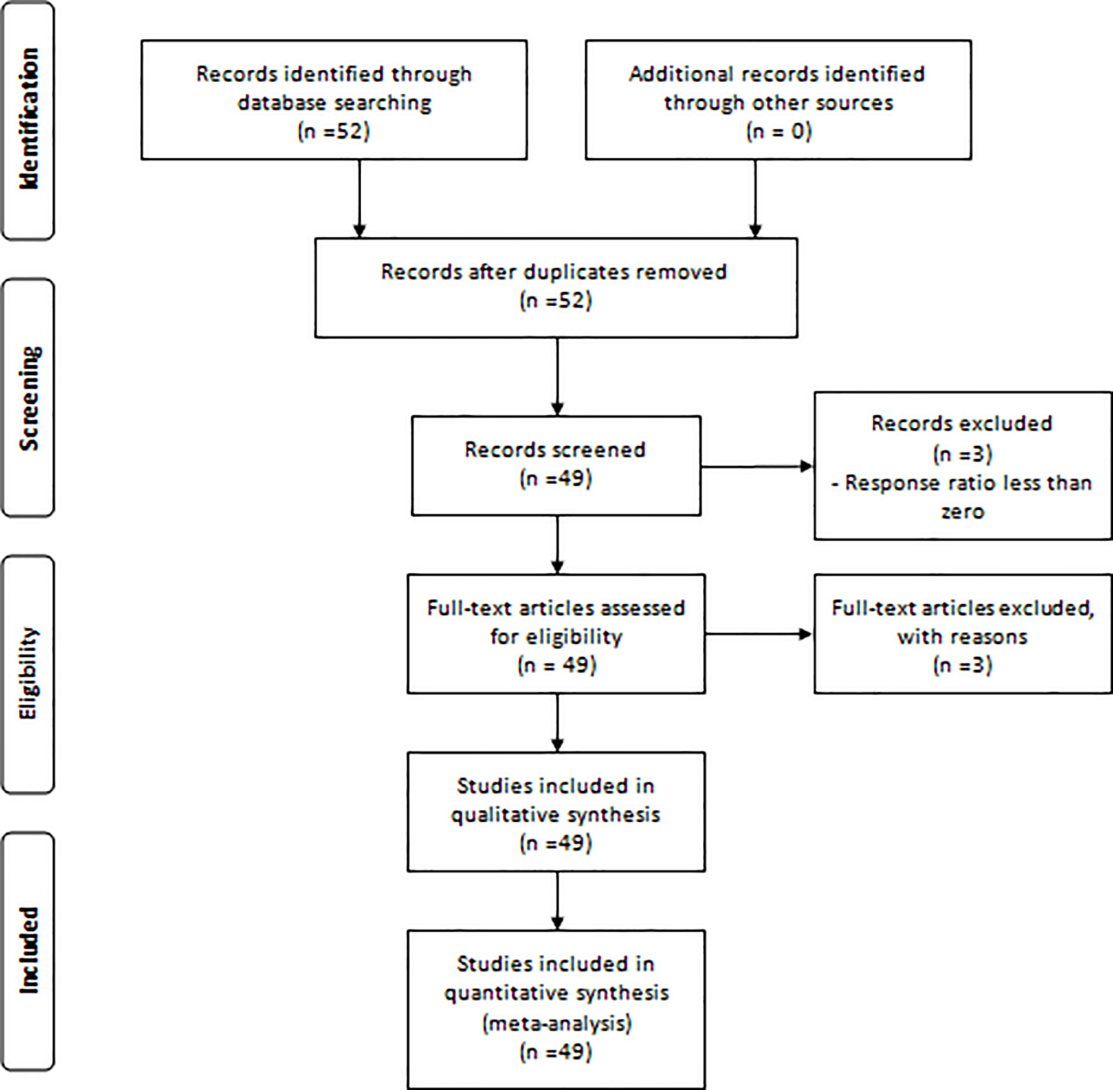
The PRISMA (preferred reporting items for system review and meta-analysis) guidelines used for the collection and meta-analysis of data.

We conducted a literature survey of peer-reviewed papers published before December 2016 that reported the effects of NT/RT on both CH_4_ and N_2_O emissions using Google Scholar and ISI-Web of Science. The preferred reporting items for system review and Meta-analysis (PRISMA) guidelines (Fig 1) have been followed for data collection and analysis. The keywords used in literature search were “soil tillage”, “no-tillage”, “reduced tillage”, “CH_4_”, “N_2_O”, and “greenhouse gas emission”. The following 3 criteria were used to select appropriate paired experiments: (1) the NT/RT and CT plots must be conducted at the same site with the same crop, agronomic management options (e.g., fertilizer, irrigation,), and experiment duration; (2) CH_4_ and N_2_O fluxes were both measured by using statistic chamber methods in field conditions for an entire crop growing season; and (3) the N application rate, crop straw returning methods, experimental duration, and water management practices were clearly recorded. Forty-nine studies including 196 comparisons (Table 1) were collected according to these criteria. The experiment sites covered 7 countries (USA, Brazil, China, Japan, Mexico, Philippines and Spain). The land use types in selected studies were primarily paddy field and upland. The crops included rice, wheat, maize, soybeans, barley, oats, and vetch. RT consisted of rotary tillage, zone tillage, shallow plowing, precision tillage, and subsurface tillage. The detailed information of selected studies and collected data is listed in the support information (Table A in S1 Appendix).

**Table 1:** The studies used in the meta-analysis to evaluate the effectiveness of NT/RT on CH_4_ and N_2_O emissions.

ID	Crop	Country	Number of comparisons	Tillage methods	Reference	ID	Crop	Country	Number of comparisons	Tillage methods	Reference
1	Wheat, pea	China	4	NT	[25]	26	Rice	China	2	RT	[45]
2	Rice	China	4	NT, RT	[26]	27	Rice	China	2	NT	[12]
3	Rice	China	2	NT	[27]	28	Rice	Brazil	1	NT	[46]
4	Rice	China	8	RT	[28]	29	Rice	Japan	2	NT, RT	[6]
5	Rice	China	4	RT	[7]	30	Maize	America	2	NT	[47]
6	Wheat	Spain	2	NT, RT	[9]	31	Wheat	Japan	3	RT	[48]
7	Wheat, maize	China	6	NT, RT	[29]	32	Wheat	America	4	NT, RT	[16]
8	Rice	China	8	NT	[30]	33	Wheat	America	2	NT, RT	[49]
9	Wheat	China	1	NT	[31]	34	Barley, vetch	Spain	4	NT, RT	[50]
10	Barley, Soybean	Japan	8	NT	[32]	35	Wheat, Maize	China	6	NT, RT	[51]
11	Rice	Philippines	2	NT	[33]	36	Wheat	China	1	RT	[52]
12	Barley	America	16	NT	[11]	37	Rice	China	2	NT	[53]
13	Rice	Brazil	3	NT	[34]	38	Wheat, pea	China	6	NT	[54]
14	Maize	China	4	NT, RT	[35]	39	Rye	Spain	1	NT	[55]
15	Wheat	China	4	NT	[36]	40	Rice	Spain	3	NT	[56]
16	Rice	China	2	NT, RT	[37]	41	Rice	China	16	NT	[57]
17	Wheat	China	2	NT	[38]	42	Rice	China	2	NT	[23]
18	Wheat	China	8	NT, RT	[39]	43	Rice	India	2	NT	[58]
19	Rice	China	2	RT	[40]	44	Rice	Japan	4	RT	[59]
20	Wheat	China	4	NT, RT	[41]	45	Vegetable	Japan	5	NT	[60]
21	Barley	America	6	NT	[13]	46	Vetch	Spain	2	NT, RT	[61]
22	Maize	Mexico	2	RT	[42]	47	Bioenergy crop	China	2	NT	[62]
23	Oat	China	3	NT	[43]	48	Wheat, pea	China	4	NT	[63]
24	Wheat, pea	China	4	NT	[44]	49	Rice	China	1	NT	[64]
25	Maize, soybean	America	8	NT, RT	[10]						

The results of CH_4_ and N_2_O emissions were converted into global warming potential (GWP) by multiplying the 100-year radiative forcing potential coefficients to CO_2_ (25 and 298 used for CH_4_ and N_2_O, respectively). Considering that upland and paddy fields showed a great difference in CH_4_ and N_2_O emissions, we examined the effect of NT/RT on GHG emissions for these two land use types separately.

Five agronomic practices, including cropping system, residue management, N split, irrigation, and tillage duration, were analyzed. Cropping system was categorized into single crop monoculture system and double crops rotation system for upland, and rice-upland crop rotation and double rice for paddy field, referred to the crop sequences in a whole year. Residue management practices were categorized as crop straw remove and return. We further analyzed the effects of N split methods on the effect of NT/RT. N split methods were categorized into four groups based on the percentage of basal N fertilizer (P_BN_ = 100%, 50% < P_BN_ < 100%, P_BN_ = 50%, and P_BN_ < 50%; P_BN_ = basal N rate / total N rate *100). Irrigation practices were divided into rain-fed and irrigation for uplands, and continuous flooding and intermittent irrigation for paddy fields. Tillage duration was divided into three levels: short-term 1–5 years; medium-term 5–10 years; and long-term >10 years.

### Data analysis

The response ratio (R) was used to compare the CH_4_, N_2_O and overall GWP under NT/RT and CT. The natural log of R was used as the effect size, which was calculated by following equation:

$$LnR=Ln(\frac{X_{NT/RT}}{X_{CT}})$$ (1)

Where, X_NT/RT_ and X_CT_ are the amounts of CH_4_, N_2_O and overall GWP under NT/RT and CT, respectively.

This meta-analysis was performed by using a nonparametric weighting function and the confidence intervals (CIs) were generated by using bootstrapping [14]; because only 22.4% of selected studies reported the standard deviation or error of CH_4_, N_2_O and GWP. Effect size was weighted by the number of experiment replicates and the number of CH_4_ and N_2_O flux measurements per month.

W=n*f (2)

Where, W is the weight factor, n is the number of experiment replicates; and f is the number of CH_4_ and N_2_O flux measurements per month. This weighting approach assigned more weight to field experiments that were well replicated.

The mean effect size was calculated from lnR of individual studies by:

$$M=\left(EXP(\frac{\sum \left(lnR(i)*w(i)\right)}{\sum w(i)})-1\right)*100$$ (3)

Where, w(i) is the weighting factor estimated by formula (2). To ease interpretation, the mean effect size was back-transformed and reported as the percent change of NT/RT relative to that of CT.

The Mean effect size, 95% confidence intervals (CIs), group heterogeneity and publication bias were calculated by MetaWin 2.1[65]. The random-effects model was used in the calculation of mean effect sizes, based on the assumption that random variation in GHG emissions occurred between observations. The 95% CIs around mean effect sizes were calculated by using bootstrapping with 4999 iterations [66]. The mean effect sizes were considered to be significantly different if their 95% CIs did not overlap. P-values for differences between categories of studies (Table 2) were calculated using resampling tests [14]. The publication bias was checked using Rank Correlation Test method (Kendall's tau and Spearman rank-order correlation). For the categories existing publication bias, a bias corrected CI was used instead of bootstrap CI.

**Table 2:** The group heterogeneity of categorical variables (NT/ RT, P-value).

	Categorical variable	CH_4_	N_2_O	GWP
Upland	Crop rotation	0.157/ 0.643	**0.005**/ 0.193	0.136/ **0.012**
	residue management	0.606 / 0.751	0.310 / 0.797	0.392 / 0.508
	N split	0.907/ 0.140	0.213/ 0.569	0.133/ 0.293
	Irrigation	0.446/ 0.082	0.698/ 0.564	0.690/ 0.178
	tillage duration	0.293/ **0.008**	**0.006**/ 0.619	**<0.001**/ 0.922
Rice paddy	Crop rotation	0.330/ 0.353	0.053/ 0.584	0.073/ 0.232
	residue management	0.137/ 0.055	0.939/ 0.166	0.097/ **0.015**
	N split	**0.003**/ 0.424	0.943/ 0.492	**0.005**/ 0.544
	Irrigation	**0.036**/ NA	0.076/ NA	**0.010**/ NA
	tillage duration	0.231/ **0.004**	0.148/ 0.616	0.101/ **0.019**

P-values in bold indicate significance (P < 0.05). “NA” means not available

## Results and discussion

### Overall effect

**Fig 2:**
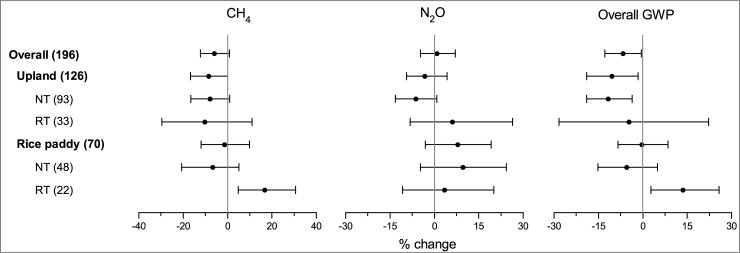
The overall effects of reduced tillage system on CH_4_, N_2_O and overall GWP.

**Fig 3:**
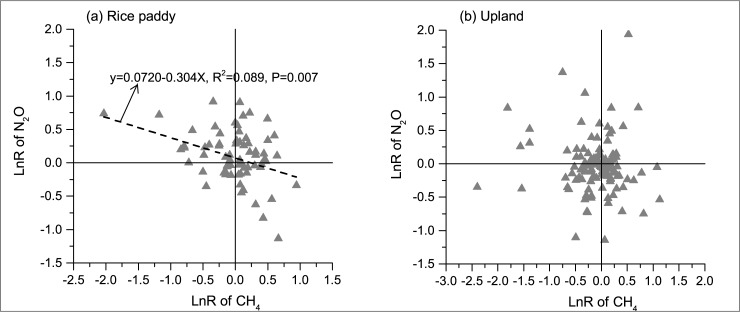
The relationship of the LnR of NT/RT on CH_4_ and N_2_O emissions in paddy and upland fields.

Overall, reduced tillage system (NT/RT) did not exhibit significant effects on CH_4_ and N_2_O emissions as compared with CT (Fig 2). However, their effects on the overall GWP of CH_4_ and N_2_O emissions was marginally significant. The overall GWP was mitigated by 6.6% by NT/RT as compared with CT. The performance of NT/RT depended on land use type and tillage methods. In rice paddies, a trade-off relationship existed in the effects of NT/RT on CH_4_ and N_2_O emissions (Fig 3(A)). NT tended to mitigate the CH_4_ emission, whereas it increased the N_2_O emission, resulting in no significant impact on the overall GWP in rice paddies (Fig 2). However, RT significantly increased the CH_4_ emission and overall GWP compared with CT. The poor performance of RT in the inhibition of CH_4_ emission was possibly attributed to its weaker effect on reducing CH_4_ production than that of CT and weaker effect on increasing CH_4_ oxidation than that of NT. CT incorporated crop residue into deeper soil than RT, reducing the decomposition of these residues through the protection of the soil matrix [7]. Lower soil disturbance and a shallower CH_4_ oxidation zone for NT than RT were conducive to improving CH_4_ oxidation [37, 48].

Unlike that of rice paddies, upland soil usually absorbed CH_4_ from the atmosphere through the microbial process of CH_4_ oxidation by methanotrophs [67]. The effect of NT/RT on CH_4_ uptake did not show an obvious relationship with its impact on N_2_O emission (Fig 3(B)). The overall GWP was reduced 11.6% by NT as compared with CT (Fig 2). Whereas the effects of RT on CH_4_ uptake, N_2_O emission and overall GWP were not significant.

### Impact of cropping system

**Fig 4:**
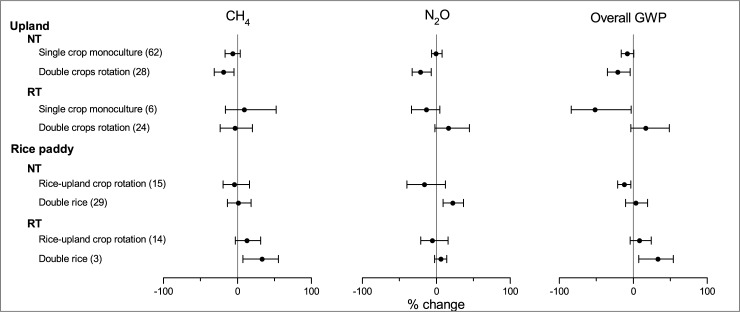
Impact of cropping system on the effects of NT and RT on CH_4_, N_2_O and overall GWP.

NT significantly reduced CH_4_ uptake, N_2_O emission, and overall GWP by 18.4%, 21.0% and 20.8% under the double crops rotation system, respectively, whereas its effects was not significant under the single crop monoculture system (Fig 4). The effects of cropping systems on CH_4_ and N_2_O emissions after adopting NT may primarily attribute to the variability in the quantity of aboveground crop residues and roots in soil profile. Increasing in cropping frequency and crop diversity, such as double crops rotation, can produce more residues and roots than that of single crop monoculture system. Most of the crops in the double crops rotation system of this analysis were cereals crops (such as maize, wheat, and barley) with high C:N ratio. The decomposition of crop residues with high C:N ratio could stimulated microbial N immobilization in soil, thus reduce the available N for N_2_O production [68]. Additionally, the decomposition of crop residues also consumed sizable O_2_ in soil pores, which may inhibit the CH_4_ oxidation [69]. RT significantly reduced the overall GWP of CH_4_ and N_2_O by 20.8% under the single crop monoculture system, as compared with CT (Fig 4). However, its effect on the overall GWP was not significant under the double crops rotation system. The upland field was usually tilled once in one year under the monoculture system; but usually two times per year under the rotation system. Less tillage operation could reduce the disturbance to methanotrophic microbes and enhance CH_4_ uptake [9]. Less tillage operation could also prevent soil aggregates and inhibit organic N mineralization, which is beneficial to the mitigation of N_2_O production [68].

As in the paddy fields, NT significantly reduced the overall GWP by its cumulative abatement on CH_4_ and N_2_O emissions (Fig 4) under the rice-upland crops rotation system; whereas its effect was not significant on the overall GWP under double rice system. A previous study has reported that soils with higher water contents during the annual crop growing season produced more CH_4_ because of their higher methanogenic populations and activities [70]. The annual flooding time was less for the rice-upland crop rotation system than the double rice system. The rotation of upland crop could improve the soil permeability, aerobic condition and methanogenic activities, which may enhance the inhibition effect of NT on the CH_4_ and N_2_O emissions [28, 71]. NT significantly increased the N_2_O emission under the double rice system. This was possibly because of the difference in temperature between late rice and winter upland crops. Late rice was commonly planted in summer, whereas winter upland crops were planted in fall. Higher temperature with surface applied N fertilizer may stimulate the N_2_O emission [57]. RT significantly increased the CH_4_ emission and the overall GWP under the double rice system. But it should be noted that only three comparisons from two studies were included for this category. The existence of publication bias was suggested by Spearman Rank-Order Correlation.

### Impact of crop straw management

**Fig 5:**
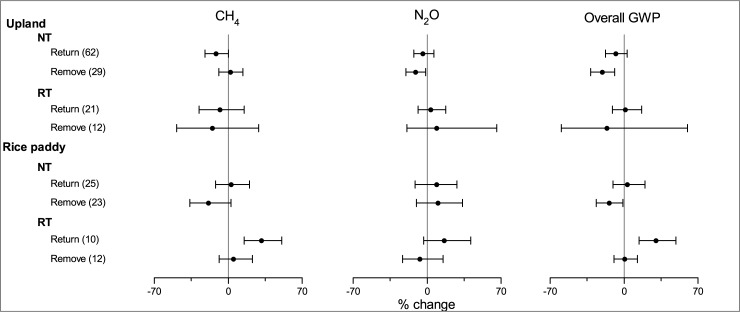
Impact of crop straw management on the effects of NT and RT on CH_4_, N_2_O and overall GWP.

NT significantly reduced the N_2_O emission and the overall GWP by 10.9% and 20.4% as compared with CT, respectively, when crop straw was removed (Fig 5). However, when the crop straw was returned, the effect sizes of NT on CH_4_ uptake, N_2_O emission, and overall GWP were not significant. Crop straw has direct and indirect positive effects on N_2_O production. The decomposition of crop straw directly provided substrate C and N for nitrifiers and denitrifiers, which may stimulate the N_2_O production in soil [68]. Indirectly, the returned crop straw was commonly mulched on the soil surface in the NT field, which could reduce soil water evaporation and conserve rainwater in situ, resulting in enhanced soil moisture [72]. High soil moisture promotes N_2_O production and inhibits CH_4_ oxidation by reducing gas diffusion [73–74]. Therefore, crop straw return may weaken the effects of NT on the mitigation of N_2_O and CH_4_ emissions.

As in paddy fields, NT significantly reduced the overall GWP by 20.4% when crop straw was removed; but did not affect the overall GWP when crop straw was returned (Fig 5). RT significantly increased the CH_4_ emission and overall GWP when crop straw was returned; whereas it did not increase the overall GWP when crop straw was removed. The effect of soil tillage on CH_4_ emission in paddy field was mostly determined by its influence on straw decomposition, which provides abundant C substrate for CH_4_ production [75]. Soil tillage determined the vertical distribution of crop straw in the soil profile. RT usually mixed straw into the surface soil (5–10cm depth); whereas plowing in CT buried the crop straw into the deeper soil layer (10–15cm), which could reduce access of these residues by microbes by the protection of soil matrix [28]. Thus, RT with crop straw return might facilitate crop straw decomposition into intermediate products that serves as substrates for methanogens [67], resulting in producing higher CH_4_.

### Impact of N split

**Fig 6:**
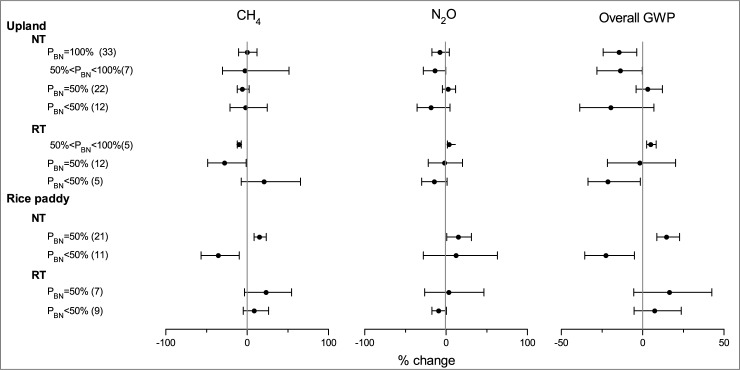
Impact of N split application on the effects of NT and RT on CH_4_, N_2_O and overall GWP.

Split application of N fertilizer is an important practice to synchronize nutrient supply with crop demand and reduce N loss to the environment. Tillage directly affected the vertical distribution and transformation of basal N fertilizer. The effectiveness of NT/RT on CH_4_ and N_2_O emissions may influenced by the percentage of basal N fertilizer (P_BN_). As shown in Fig 6, when the P_BN_ = 100%, NT significantly reduced the overall GWP by 14.4% in upland, as compared with CT. When 50% < P_BN_ < 100%, the overall GWP was marginally significantly mitigated by 13.6% by NT in upland. When P_BN_ < or = 50%, NT did not significantly affect CH_4_ uptake, N_2_O emission, and the overall GWP. This can be explained by two possible reasons. Firstly, basal N fertilizer is commonly applied with tillage operation; whereas topdressing N fertilizer is usually applied with irrigation or precipitation in uplands. Soil tillage had a greater effect on the microbial process of the basal N fertilizer than top dressing N fertilizer. Thus, high P_BN_ may intensify the inhibition effect of NT on N_2_O emission. Secondly, a large amount of field studies has reported that reducing the ratio of basal N and increasing the ratio of topdressing N enhanced plant N recovery and the N use efficiency [76]. Less P_BN_ could improve the synchronization of crop demand with N supply and inhibit N_2_O emission. Therefore, the better synchronization of crop demand with N supply may weaken the inhibition effect of NT on N_2_O emission. RT significantly reduced CH_4_ oxidation and enhanced N_2_O emission when the percentage of P_BN_ was between 50% and 100% in uplands (Fig 6). The overall GWP was significantly enhanced by 5.1% by RT. However, the existence of publication bias was suggested by Spearman Rank-Order Correlation. When P_BN_ was decreased to less than 50%, RT significantly mitigated the overall GWP because of the increase in CH_4_ uptake and the reduction in N_2_O emission. These results indicated that high P_BN_ (> 50%) with NT and low P_BN_ (< 50%) with RT benefited the GHG mitigation in upland fields.

As in paddy fields, NT significantly mitigated the overall GWP by 22.4% when P_BN_ < 50%; whereas increased the overall GWP by 14.9% when P_BN_ = 50%, which was different from that of uplands (Fig 6). The effectiveness of NT on overall GWP was largely determined by its impact on CH_4_ emission in paddy fields. The surface application of basal N fertilizer under NT could have either positive or negative effects on CH_4_ emission. On one hand, the production of CH_4_ mostly occurred at the surface layer because of the surface application of basal N fertilizer under NT, which benefited the diffusion of CH_4_ from the soil to the atmosphere [21]. On the other hand, a shallow CH_4_ production zone may also benefit CH_4_ oxidation, because the interface between water and soil was a main CH_4_ oxidation zone [37]. The integrated effect was possibly determined by the basal N application rate. The average basal N rate were 45 and 90 kg N ha^-1^ for the groups of P_BN_ < 50% and P_BN_ = 50%, respectively. We speculated that, at low P_BN_, rice uptake might outcompete the microbial process of CH_4_ production because of the limited N source [77]; and the capability of CH_4_ oxidation was possibly higher than CH_4_ production because of the insufficiency available N for Methanogenic bacteria. Thus, NT inhibited CH_4_ emission. With the P_BN_ increased to 50%, the N supply for Methanogenic bacteria was less serve, and the abundant N supply stimulated CH_4_ production and inhibited CH_4_ oxidation by suppressing Methanotrophs and switching substrates from CH_4_ to ammonia [78–79]. Additionally, the shallow CH_4_ production zone under NT may enhance the flux of CH_4_ from soil to atmosphere. Thus, NT significantly increased the CH_4_ emission under high basal N application rates.

### Impact of irrigation

**Fig 7:**
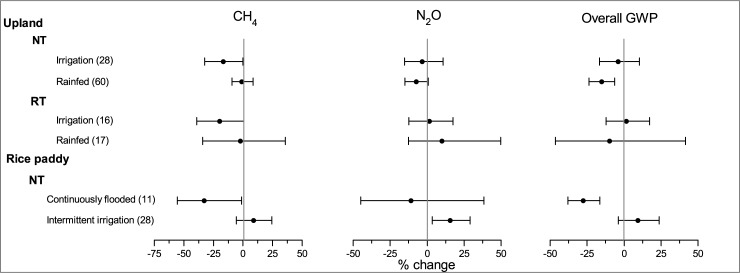
Impact of irrigation on the effects of NT and RT on CH_4_, N_2_O and overall GWP.

As shown in Fig 7, NT significantly reduced the overall GWP by 16.7% under rain-fed condition as compared with CT. The effect of NT on the overall GWP was not significant under irrigation option. NT improved soil structure, which increased the gas diffusivity and improved the tendency of the formation of aerobic microsites, and therefore increased CH_4_ oxidation and inhibited N_2_O emission [80–81]. The irrigation options in selected studies were all flooding irrigation, which may weaken the effectiveness of NT by increasing soil water and decreasing the aerobic condition in soil profile [13].

As in paddy fields, NT significantly mitigated CH_4_ emission and overall GWP in continuously flooded paddy field as compared with CT (Fig 7); whereas it did not produce a significant effect on CH_4_ and overall GWP in intermittently irrigated paddy fields as compared with CT. Continuous flooding provided a sufficient anaerobic environment for CH_4_ production in paddy fields. Thus, the labile C availability and CH_4_ oxidation capacity were two important factors controlling the total CH_4_ emission amount in flooded paddies. A previous field study showed that NT reduced the volume fraction of large soil pores, which was conducive to the prevention of the decomposition of soil organic matter [12]. Additionally, most of the crop residue and inorganic N fertilizer were placed on the soil surface in NT paddy fields, which enhanced the CH_4_ oxidation and prevented the anaerobic decomposition of organic matter because of the high O_2_ content in the soil-water interface [6, 37]. Therefore, NT was conducive to inhibition of CH_4_ emission under continuous flooded field conditions. As in intermittent irrigated field, the water usually drained out and maintained the dry-wet alternation after the rice tillering stage, which greatly increased the aerobic periods and mitigated the CH_4_ emission as compared with that of continuous flooding [82]. Thus, intermittent irrigation may weaken the effect of NT on the inhibition of CH_4_ emission.

### Impact of tillage duration

**Fig 8:**
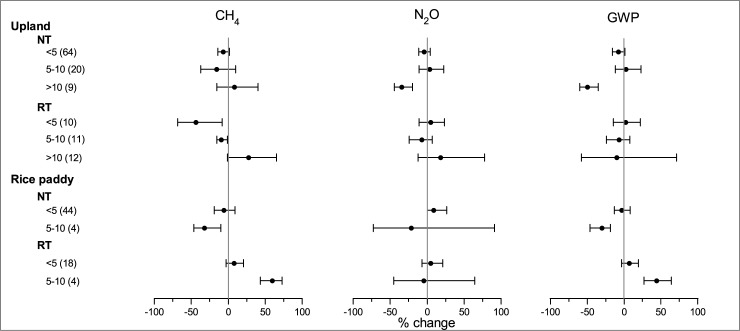
Impact of tillage duration on the effects of NT and RT on CH_4_, N_2_O and overall GWP.

NT only significantly mitigated N_2_O emission and overall GWP under long-term duration (>10 years) in uplands (Fig 8), which is consistent with previous studies [14, 83]. Long-term adoption of NT can improve soil structure and therefore is conducive to the enhancement of CH_4_ uptake and inhibition of N_2_O emission [81]. The inhibition effect of RT on overall GWP showed a trend that increase with tillage duration. However, its effectiveness was not significant because of wide variance.

As in paddy field, NT did not exhibit significant effects on CH_4_ and overall GWP under short-term duration (<5 years); whereas it significantly reduced CH_4_ and overall GWP under medium-term duration (5–10 years) (Fig 8). The existence of publication bias for the category of 5–10 years was suggested by Spearman Rank-Order Correlation. Based on a five-year field experiment, Kim et al. [22] reported that NT effectively reduced the CH_4_ emission in the 1^st^ and 2^nd^ years, but increased the CH_4_ emission in the 5^th^ year, because of increased soil organic carbon (SOC) content, as compared with CT. The contrasting results were possibly attributed to the difference in the cropping practices. The cropping system in the study of Kim et al. [22] was mono-rice with winter fallow; and the crop residue was placed in the field after rice harvest and would have completely decomposed under aerobic conditions in winter [84–85]. The SOC content was possibly the primary factor determining the CH_4_ production in the following rice cropping season. Thus, NT increased the SOC resulting in enhanced CH_4_ emission. As in the selected studies [86–87] in this analysis, the cropping systems were rice-upland crops rotation and the upland crop residue applied preceding the rice cropping provided an abundant substrate for CH_4_ production. The effect of NT on CH_4_ emission was possibly controlled by its impact on CH_4_ oxidation. Thus, continuous adoption of NT may facilitate the CH_4_ oxidation and significantly reduce the CH_4_ emission. These results indicated that the temporal effect of NT in rice paddies might highly depend on the cropping system. However, the long-term experiment of NT in paddy fields was still limited. The number of long-term experiments conducted in paddy field was far less than that in upland. More field studies are needed to investigate the temporal effect of NT on GHG emission in paddy field under different agricultural practices.

## Summary

NT/RT significantly reduced the overall GWP of CH_4_ and N_2_O emissions by 6.6% as compared with CT. The effectiveness of NT/RT depended on tillage methods, land use type, and agricultural practices. The suggested practices for NT to reduce the GHG emission were crop rotation and straw remove both in upland and paddy field, P_BN_>50% and rainfed in upland, while P_BN_<50% in paddy field. RT was less effective on the mitigation of GHG emission than NT. RT significantly enhanced the CH_4_, N_2_O or overall GWP under several practices, such as double rice system and crop straw returned in paddy field, and P_BN_>50% in upland. Only single crop monoculture facilitated RT to reduce the overall GWP.

## Supporting information

S1 Appendix The detailed information of selected studies. (XLSX)

S2 Appendix Literature search strategy. (DOCX)

S3 Appendix PRISMA checklist. (DOC)
