# Supplementary material for: Impact of agronomy practices on the effects of reduced tillage systems on CH4 and N2O emissions from agricultural fields: A global meta-analysis
Source: PLoS One. 2018 May 21;13(5):e0196703. doi: 10.1371/journal.pone.0196703 (PMC5962074; doi:10.1371/journal.pone.0196703)
Supplement: S2 Appendix — (DOCX) [file pone.0196703.s002.docx]

**Literature search strategy:**

The keywords used in literature search were “soil tillage”, “no-tillage”, “reduced tillage”, “CH_4_”, “N_2_O”, and “greenhouse gas emission”.

The literature search was conducted in the ISI-Web of Science and Google Scholar.

The literature search strategy were:

1. “soil tillage” and “CH_4_” within all years
2. “soil tillage” and “N_2_O” within all years
3. “soil tillage” and “greenhouse gas emission” within all years
4. “no-tillage” and “CH_4_” within all years
5. “no-tillage” and “N_2_O” within all years
6. “no-tillage” and “greenhouse gas emission” within all years
7. “reduced tillage” and “CH_4_” within all years
8. “reduced tillage” and “N_2_O” within all years
9. “reduced tillage” and “greenhouse gas emission” within all years
